# Remote Monitoring of Inhaled Bronchodilator Use and Weekly Feedback about Asthma Management: An Open-Group, Short-Term Pilot Study of the Impact on Asthma Control

**DOI:** 10.1371/journal.pone.0055335

**Published:** 2013-02-27

**Authors:** David Van Sickle, Sheryl Magzamen, Shaun Truelove, Teresa Morrison

**Affiliations:** 1 Department of Population Health Sciences, University of Wisconsin School of Medicine and Public Health, Madison, Wisconsin, United States of America; 2 Air Pollution and Respiratory Health Branch, National Center for Environmental Health, Centers for Disease Control and Prevention, Atlanta, Georgia, United States of America; Tehran University of Medical Sciences, Iran (Republic of Islamic)

## Abstract

**Objective:**

Adequate symptom control is a problem for many people with asthma. We asked whether weekly email reports on monitored use of inhaled, short-acting bronchodilators might improve scores on composite asthma-control measures.

**Methods:**

Through an investigational electronic medication sensor attached to each participant's inhaler, we monitored 4 months' use of inhaled, short-acting bronchodilators. Participants completed surveys, including the Asthma Control Test^TM^ (ACT), to assess asthma control at entry and monthly thereafter. After the first month, participants received weekly email reports for 3 months. The reports summarized inhaled bronchodilator use during the preceding week and provided suggestions derived from National Asthma Education and Prevention Program (NAEPP) guidelines. Paired t-tests and random-effects mixed models were implemented to assess changes in primary asthma endpoints.

**Results:**

Thirty individuals participated in the 4-month study; 29 provided complete asthma control information. Mean age was 36.8 years (range: 19–74 years); 52% of respondents were female. Mean ACT scores were 17.6 (Standard Deviation [SD]  = 3.35) at entry and 18.4 (SD = 3.60) at completion of the first month. No significant difference appeared between ACT values at entry and completion of the first month (p = 0.66); however, after participants began receiving email reports and online information about their inhaler use, mean ACT scores increased 1.40 points (95% CI: 0.61, 2.18) for each subsequent study month. Significant decreases occurred in 2-week histories of daytime symptoms (β = −1.35, 95% CI: −2.65, −0.04) and nighttime symptoms (β = −0.84, 95% CI: −1.25, −0.44); no significant change in activity limitation (β = −0.21, 95% CI: −0.69, 0.26) was observed. Participants reported increased awareness and understanding of asthma patterns, level of control, bronchodilator use (timing, location) and triggers, and improved preventive practices.

**Conclusions:**

Weekly email reports and access to online charts summarizing remote monitoring of inhaled bronchodilator frequency and location were associated with improved asthma control and a decline in day-to-day asthma symptoms.

## Introduction

Despite improved understanding and the development of new medications, asthma remains a substantial and costly public health problem [1]. Each year in the United States, 1.75 million asthma-related emergency department visits and 456,000 asthma hospitalizations occur [2], with overall costs of $55 billion [1]. While proper treatment could prevent the majority of exacerbations, numerous studies suggest that a majority of people with asthma do not have adequate control of their disease [3], [4].

National Asthma Education and Prevention Program and Global Initiative for Asthma clinical practice guidelines recommend doctors monitor whether treatment is controlling symptoms and improving quality of life [5], [6]; however, physicians lack ways to objectively assess how well patients manage asthma symptoms between visits, and often underestimate the frequency and severity of patients' symptoms [7]. In addition, the majority of patients with uncontrolled asthma report that their disease is well-managed [3].

Questionnaires that capture composite asthma-control measures have become popular tools to help patients and their physicians determine level of control [7]. These questionnaires ask patients to recall and report over some period of time symptom frequency, activity level and restriction, and inhaler usage. Underlying these instruments is evidence that symptom frequency and bronchodilator use are important indicators of asthma control, of current impairment, and of future risk of worsening asthma [6], [7].

This pilot study's goals were 1) to investigate the use of a device to monitor objectively the time and location of inhaled bronchodilator use as a measure of asthma control, and 2) to determine whether information about inhaled bronchodilator use and feedback on asthma control via weekly email reports was associated with improved scores on composite measures of asthma control.

## Methods

### Ethics Statement

The study was reviewed and approved by the University of Wisconsin Health Sciences institutional review board (protocol H-2007-0291) and all participants signed informed consent documentation.

### Participants

We recruited adults (aged >18 years) from a variety of clinical and community settings on a rolling basis between February and April 2009. These adults had a diagnosis of asthma and used a prescribed inhaled short-acting beta-agonist (SABA). By the end of August 2009, all participants had participated for 4 months. At entry, participants completed questionnaires to collect data on their demographic and clinical characteristics, including asthma history, then-current status, and asthma management such as medical treatments, perceived triggers, healthcare utilization, and level of control using the Asthma Control Test^TM^ (ACT) [8].

**Figure 1:**
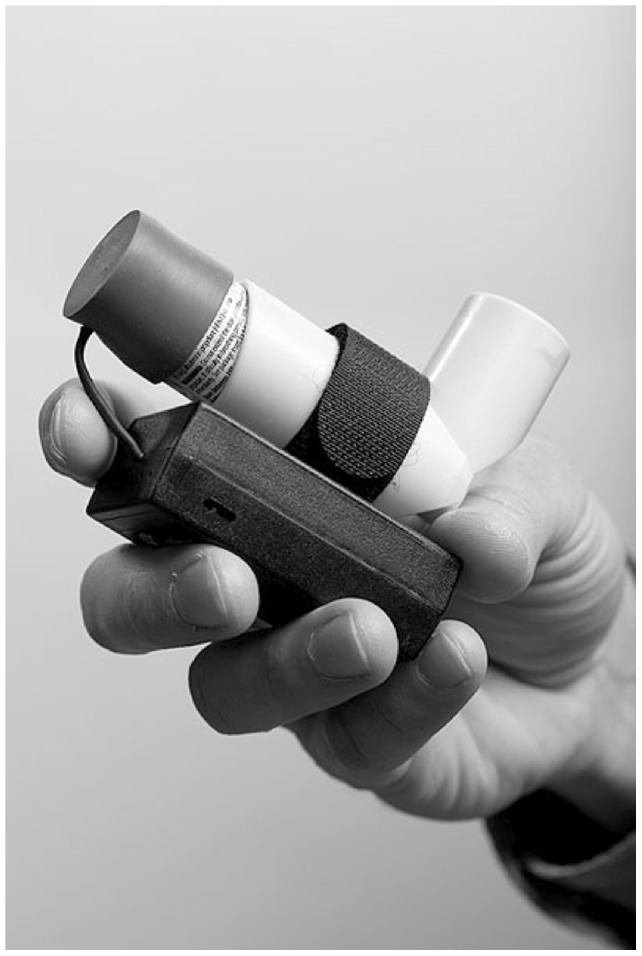
GPS-enabled inhaler sensor as attached to inhaled albuterol.

We collected data about the time and location of use of inhaled short-acting bronchodilators. This involved using a small and portable investigational electronic medication sensor that is attached to an existing inhaler housing by means of a hook-and-loop fastener strap (Figure 1). This allows the sensor to be readily transferred to a new inhaler when the medication is refilled. When the sensor was attached to an inhaler, an actuation detection assembly placed on the end of the medication canister monitored the use of the inhaler and used a global positioning system (GPS) receiver to determine the time and location of the inhaler whenever it was used. Design verification and systematic bench and human testing demonstrated that the sensor accurately and reliably detected actuation of the inhaler and that expected functioning was not sensitive to a precise method of attachment. Participants were asked to keep the sensor attached to their inhaled short acting bronchodilator for all 4 months of the study.

The device also contained equipment to communicate via a wireless network and supporting electronics, including a simple user interface, a power switch, and a rechargeable battery. The device obtained and wirelessly communicated medication event information in real-time to a remote server. It did not require that the patient (or any other person) participate in obtaining or transmitting the information. Because the routine dose of some SABAs can vary from one to multiple inhalations, the device was not designed to record each inhalation of medication but rather to capture information about the occasion of medication use, following American Thoracic Society/European Respiratory Society guidance [7].

After completing the first month of the study, participants began to receive weekly e-mail reports displaying maps and charts of their usage. They also received access to an online interface with similar features. This online interface allowed participants to indicate when an inhaler was used prophylactically before exercise. The weekly e-mail reports included the time and location for each inhaled bronchodilator use and summarized use for the preceding week. Participants received a basic assessment of their asthma control based on the number of days of use in the preceding week, the timing of those events, and on thresholds from the National Asthma Education and Prevention Program (NAEPP) guidelines. Participants also received simple advice derived from the NAEPP guidelines about how to improve asthma self-management (Appendix S1) [7].

At the conclusion of each month, and on conclusion of the study in August 2009, participants completed questionnaires designed to detect any changes in the four primary study outcome measures: the Asthma Control Test^TM^ score and 2-week histories of activity limitation and days and nights with asthma symptoms. A score ≤19 (of 25) on the Asthma Control Test^TM^ was considered to indicate uncontrolled asthma. We collected 2-week histories of asthma symptoms and activity limitation to capture continuous estimates of outcome measures in addition to the ordinal responses gathered by the Asthma Control Test^TM^. Exit questionnaires also assessed participant feedback that included the utility of the electronic communications, perceived changes in asthma awareness, and modifications in day-to-day asthma management.

### Statistical Tests

We used pairwise t-tests to evaluate the null hypothesis that there was no change in the four primary study endpoints (Asthma Control Test^TM^ score; two-week histories of activity limitation and days and nights with asthma symptoms) while participants were blinded to data collected by the medication sensor in the first month of participation. Linear mixed effects models with a random effect for subject assessed changes in primary asthma endpoints across the three subsequent months of enrollment (at the completion of the first month, the second month, and the third month, i.e., upon exit) when participants were able to access their usage data online. Participants with one or more observations were included in the analysis.

All analyses were performed with SAS 9.2 (SAS Institute, Cary, NC). Regression diagnostics were performed using the MIXED_DX macro [9].

## Results

### Participant characteristics

A total of 33 individuals were initially recruited for the study and 29 provided complete information on asthma control at entry. Table 1 presents the demographic characteristics of the participants, who averaged 36.8 years of age (range: 19–74 years); 52% of respondents were female. Four (14%) reported Hispanic ethnicity. A broad range of socioeconomic backgrounds was represented, including household income and educational achievement. Persons who completed the study did not differ significantly from those who did not. In particular, there was no significant difference in the proportion of males (95% CI: −19.8%, 36.4%), in age (p = 0.80), or in years of diagnosis (p = 0.95) in the group that enrolled compared to the group that completed the study.

**Table 1:** Demographic and clinical characteristics of participants (N = 30).

Characteristic*	
Sex	
Female	52%
Mean Age, in years(range)^†^	36.8 (19–74)
Mean total inhaler events (range)^#^	39.9 (1–191)
Income, in dollars	
<50,000	41%
50,000–99,999	21%
>100,000	31%
Prefer not to say	7%
Race/ethnicity	
White	86%
Hispanic	14%
Tobacco exposure	
Current smoker	-
Ever smoker	21%
Smoker in house	7%
Education	
Completed college and above	59%
Health care utilization	
Oral steroids in past year	31%
ED visit for asthma ever	59%
Hospitalization for asthma ever	24%
Unscheduled doctor visit past year	38%
Urgent care visit past year	24%
Current treatment	
Controller medication**	59%
Main source of asthma care	
Family practice	59%
Internal medicine	7%
Specialist (allergy, pulmonology)	34%
Preventive asthma care	
Action plan	14%
Influenza vaccination last year	59%
Peak flow meter	52%
Asthma symptom diary	-

* Presented as percent unless otherwise indicated; ^†^Value missing for one participant;^ #^Information missing for three participants; -Zero value; ** Controller medication limited to any inhaled anti-inflammatory therapy.

On average, participants had longstanding asthma, with a mean time since diagnosis of 25 years (standard deviation [SD]  = 16). Approximately 62% (18/29) of study participants entered with uncontrolled asthma; mean ACT score at study entry for uncontrolled participants was 15.4 (SD = 2.11). Mean ACT score overall was 17.6 (SD = 3.35).

At study entry, participants reported 4.84 days (SD = 4.13) with asthma symptoms, 2.03 (SD = 3.35) nights with asthma symptoms, and 0.86 (SD = 1.38) days with activity limitations in the 2 weeks before entry (Table 2). More than half (62%; 18/29) of participants reported current use of anti-inflammatory therapy (e.g., an inhaled corticosteroid). There was no significant relation between report of an inhaled corticosteroid and ACT score at study entry (Fisher's exact test: p = 1.00). Approximately one-third of the participants reported receiving ≥1 course of oral steroids in the past year, and nearly 40% reported an unscheduled doctor visit in the past year (Table 1).

**Table 2:** Reported days with symptoms, nights with symptoms, and activity limitation in preceding two weeks, and Asthma Control Test^TM^(ACT) score.

	n	Mean	SD *
**Days with Sx** ^†^ **(days)**			
Entry	29	4.84	4.13
First Month^#^	21	4.95	4.43
Second Month**	18	2.77	3.56
Exit	20	2.92	2.16
**Nights with Sx (nights)**			
Entry	29	2.03	3.35
First Month	21	1.85	2.10
Second Month	18	0.55	0.98
Exit	20	0.15	0.49
**Activity Limitations (days)**			
Entry	29	0.86	1.38
First Month	21	1.14	1.62
Second Month	18	0.50	1.04
Exit	20	0.95	2.28
**Asthma Control Test^TM^ (score out of 25)**			
Entry	29	17.6	3.35
First Month	23	18.4	3.60
Second Month	18	20.1	3.66
Exit	21	21.2	3.36

* SD = standard deviation;^ †^Sx = symptoms; ^#^First Month denotes the first month that participants received weekly email reports; **Second Month denotes the second month that participants received weekly email reports.

### Observed inhaler use

Participants were enrolled for a total of 3,887 patient-days of observation between March and August 2009. Of these days, devices were turned on and available on 1,916 days –49% of patient-days. On the other days, devices were powered off or out of range of a wireless network.

On average, participants used the devices on 27% (520/1,916) of patient-days in which the devices were active, and 13% (520/3,887) of patient-days overall. During the study period, the devices reported a total of 958 medication-use events, occurring on 520 patient-days. The median number of events per day was 1.35, with a mode of 1. The percentage of days on which individual participants used bronchodilators ranged from 1% to 83%. Overall, participants who entered the study with controlled asthma (11/29) as defined by the Asthma Control Test^TM^ (score >19) used bronchodilators on 25% of the days their devices were active, compared with 37% among those with uncontrolled asthma.

### Effect of intervention

A total of 30 individuals provided information for at least one of the periodic (monthly) surveys. Results from the pairwise t-test (Table 3) indicate that from study entry to the end of the first month, while participants were blinded to their data on inhaler usage, no significant differences appeared in the ACT score (p = 0.66). In addition, no significant differences appeared in the 2-week history of daytime symptoms, nighttime symptoms, or activity limitations from the entry period to the first-month survey.

**Table 3:** Paired t-tests for difference in asthma outcomes between study entry and first month.

Outcome	n	Entry Mean	First Month Mean	Mean Difference	SD^#^	t^†^	p value
Days with Sx*	20	4.35	5.20	0.85	3.99	−0.95	0.35
Nights with Sx*	20	1.20	1.95	0.75	1.83	−1.83	0.08
Activity Limitations	20	0.75	1.20	0.45	1.76	−1.14	0.26
ACT score	22	18.18	18.41	−0.23	2.37	−0.45	0.66

* Sx = symptoms;^#^SD = standard deviation;**^†^**t =  t-statistic.

After the first month that participants received weekly email reports, mean ACT scores increased 1.5 points to 20.1 (SD = 3.66). After a second month of reports (at exit), mean ACT scores increased an additional 1.1 point to 21.2 (SD = 3.36)(Table 2). At exit, 70% of participants had improved ACT scores, 15% had no change, and 15% had worsened. Scores for the worsened patients (n = 3) declined an average of 0.9 points. Overall, at the conclusion of the study period, 75% of participants had controlled asthma (by ACT score) compared with 38% at entry.

Table 4 shows results from the repeated-measures analysis. Each month of participation following the availability of inhaler usage data through the online interface and weekly email reports resulted in a 1.40 point (95% CI: 0.61, 2.18) increase in the Asthma Control Test^TM^ score. Receipt of weekly e-mail reports and online access to asthma event history was also associated with a significant decrease in number of days with symptoms (β = −1.35, 95% CI: −2.65, −0.04) and number of nights with symptoms (β = −0.84, 95% CI: −1.25, −0.44). We did not observe a significant change in the frequency of activity limitation reported by participants. Regression diagnostics indicated that linearity assumptions were adequately met for the primary analyses.

**Table 4:** Results for repeated measures analysis of asthma outcomes per month during the intervention period (from completion of the first month to study exit), n = 30.

Outcome	Parameter Estimate	SE^#^	Lower 95%CI^†^	Upper 95% CI^†^
Days with Sx*	−1.35	0.62	−2.65	−0.04
Nights with Sx*	−0.84	0.19	−1.25	−0.44
Activity Limitations	−0.21	0.22	−0.69	0.26
ACT score	1.40	0.37	0.61	2.18

* Sx = symptoms;^#^SE = standard error;**^†^**CI = confidence interval.

On exit surveys, participants reported that the weekly email reports increased their awareness of the frequency and patterns of asthma symptoms. Participants suggested that the feedback helped improve perceptions of their asthma control and their ability to communicate to their physicians their level of control. As one participant described, “I learned that I used my inhaler more than I remember. I was able to see and relate to my doctor that my asthma is not under control.” Participants also reported that the receipt of information about the time and location where they used their inhaler helped to highlight locations and exposures to triggers that led to symptoms. “I've been more keen to note surroundings when I feel shortness of breath,” one participant said. “It opened my eyes to triggers I wasn't aware of in the past.” Some described subsequent attempts to minimize or avoid exposure. Finally, participants reported that weekly feedback helped to reinforce their use of controller medications. “I'm now using my controller medicine more regularly and on time,” one participant told us. “I noticed that my rescue inhaler use went down significantly when using a daily inhaler as well.”

Participants reported that remote monitoring and feedback proved a practical aid. All but one of the participants indicated that the weekly e-mail reports were either very or somewhat useful in improving their asthma management; overall, 80% expressed an interest in continuing to track their inhaler use.

## Discussion

The findings from this pilot study suggest that capturing data on inhaled bronchodilator use, incorporating that information with some basic asthma education, and putting it all into a simple, weekly e-mail report may be associated with improved asthma control. The majority of participants in this study experienced small, but statistically significant and steady increases in composite measures of asthma control and significant declines in the day-to-day burden of asthma symptoms. We observed a mean per month increase of 1.4 points in ACT score, or 2.8 points overall, slightly less than the Minimal Important Difference of 3 points [10]; however, no plateau in improvement was observed during the study period. The availability of real-time information on the frequency and time of day of bronchodilator use allowed rapid, automated assessment of asthma control.

This study provided patients with novel feedback about the ongoing management of their asthma. Today, many patients have inappropriately low expectations for their own disease control or are unaware that more can be done to prevent attacks and day-to-day symptoms. In one recent study, more than 4 in 5 individuals with poorly controlled asthma regarded their disease as well controlled or very well controlled [3]. Others fail to voice concerns or to report troublesome symptoms. This study has demonstrated that simple interventions that use objectively collected data from daily life may provide patients with valuable information to help guide management and achieve control of their disease. These interventions can help individuals with asthma assess their level of asthma control and can provide ways to convey that information to their doctors.

A number of recent studies have demonstrated that internet- and mobile phone-based self-management interventions can improve asthma control and asthma-related quality of life [11]–[15]. As many proven asthma self-management tools such as asthma action plans and asthma symptom diaries are still not widely used [16], new methods of delivering personalized, targeted self-management materials online and via mobile phone have the potential to increase adoption and engagement, potentially benefiting many patients. This study suggests that technology and rapid feedback in the form of charts, graphs, and maps can make self-management more interesting and compelling, and can play a valuable role in developing and reinforcing better habits, particularly when integrated with a comprehensive education and self-management program. Such a system can be extended to remotely monitor the inhaler use of patients in real time. Also, it can routinely evaluate medication use against physician-established thresholds or national guidelines. Patients in need of additional review or attention could be automatically identified, and providers or family members could be contacted as necessary.

The results of this pilot study are subject to numerous limitations. In particular, this study purposefully enrolled a small convenience sample of adult volunteers with relatively uncontrolled asthma that does not represent that general population with asthma or specific asthma phenotypes. Participants in the study did not undergo reversibility studies to confirm their reported diagnosis of asthma, nor were pulmonary function studies performed to quantify the severity of airflow limitation. Given the age range of participants, it remains possible that some persons may have also had undiagnosed chronic obstructive pulmonary disease. In addition, not all participants in this study provided complete information on all questionnaire outcomes at all time points. While attrition may have introduced some bias, persons who did not provide complete information or who withdrew did not differ significantly from those who completed the study. That many individuals continued to contribute information from the electronic medication sensor even when they failed to respond to asthma control questionnaires underscores the unique potential role and value of passively collected data in the remote monitoring of asthma control.

This study was also limited by its relatively short duration and the lack of a control group to isolate the effect of time-varying covariates (such as environmental exposures) known to influence asthma. As a result, it is not possible to know if participants would have improved regardless of the intervention (due, for example, to regression to the mean or seasonal trends). Subsequent research with a randomly selected sample and a randomized and controlled protocol of greater duration is underway to isolate and assess the effect and persistence of remote monitoring and feedback on management. Stratification by level of control at entry should also strengthen the analysis and interpretation [13]. Finally, despite the positive reception from the community and the enthusiasm of participants, we found that two individuals failed to keep the device adequately charged for much of the study. This resulted in periods where no inhaler use (or absence of use) could be recorded. Fortunately, the wireless nature of the device allows us to create an accurate record of the days during which the device was operating, and this minimized potential bias.

This pilot study has identified a number of areas where additional research could improve the value of remote monitoring and patient feedback, and underscores the need for evaluation of its potential effects in a properly designed and conducted randomized-controlled trial. For example, more information is required to understand how participants interpret weekly feedback and how often they share reports with friends, family, and health care providers. Further research is also needed to determine whether providing patients with information about their patterns of inhaler use motivates them to adhere better to preventive therapy, or increases the probability that patients undertake environmental interventions or behavior changes to reduce exposures that cause attacks. Finally, we recommend additional research to identify which composite measure of asthma control, or combination of questions from available instruments, best captures current impairment and future risk when used in conjunction with information about rescue and controller medication use captured by remote monitoring devices.

Remote monitoring of inhaled bronchodilator use may also be valuable for epidemiological research and public health surveillance. By aggregating individual patterns of inhaler use, epidemiologists and public health groups can use the accumulated data to strengthen their ongoing asthma surveillance activities, improve scientific understanding of disease triggers and progression in the community, and provide a novel source of data about the local burden of disease through which to examine the response to treatment and interventions. Data can also target and evaluate interventions designed to reduce asthma-related morbidity in the community. Monitoring the timing and location of use of inhaled bronchodilators provides instant, objective data on the burden and frequency of asthma symptoms in populations. With widespread use it may also reveal clusters of asthma exacerbations and help identify new epidemiological patterns and risk factors, particularly when used as part of focused investigations.

## Conclusion

Receipt of weekly email reports with asthma management guidance and access to online materials summarizing use of inhaled bronchodilators was associated with improved measures of asthma control. Such a system has the potential to improve the recognition and treatment of poorly controlled asthma. It could also represent an important improvement in asthma management, epidemiological research, and public health surveillance.

## Supporting Information

Appendix S1 Sample weekly email report.
(TIFF)
